# Redefining pandemic preparedness: Multidisciplinary insights from the CERP modelling workshop in infectious diseases, workshop report

**DOI:** 10.1016/j.idm.2024.02.008

**Published:** 2024-02-23

**Authors:** Marta C. Nunes, Edward Thommes, Holger Fröhlich, Antoine Flahault, Julien Arino, Marc Baguelin, Matthew Biggerstaff, Gaston Bizel-Bizellot, Rebecca Borchering, Giacomo Cacciapaglia, Simon Cauchemez, Alex Barbier--Chebbah, Carsten Claussen, Christine Choirat, Monica Cojocaru, Catherine Commaille-Chapus, Chitin Hon, Jude Kong, Nicolas Lambert, Katharina B. Lauer, Thorsten Lehr, Cédric Mahe, Vincent Marechal, Adel Mebarki, Seyed Moghadas, Rene Niehus, Lulla Opatowski, Francesco Parino, Gery Pruvost, Andreas Schuppert, Rodolphe Thiébaut, Andrea Thomas-Bachli, Cecile Viboud, Jianhong Wu, Pascal Crépey, Laurent Coudeville

**Affiliations:** a Center of Excellence in Respiratory Pathogens (CERP), Hospices Civils de Lyon (HCL) and Centre International de Recherche en Infectiologie (CIRI), Équipe Santé Publique, Épidémiologie et Écologie Évolutive des Maladies Infectieuses (PHE3ID), Inserm U1111, CNRS UMR5308, ENS de Lyon, Université Claude Bernard Lyon 1, Lyon, France; b South African Medical Research Council, Vaccines & Infectious Diseases Analytics Research Unit, Faculty of Health Sciences, University of the Witwatersrand, Johannesburg, South Africa; c New Products and Innovation (NPI), Sanofi Vaccines (Global), Toronto, Ontario, Canada; d Department of Mathematics and Statistics, University of Guelph, Guelph, Ontario, Canada; e Fraunhofer Institute for Algorithms and Scientific Computing (SCAI), Department of Bioinformatics, Schloss Birlinghoven, Sankt Augustin, Germany; f University of Bonn, Bonn-Aachen International Center for IT (b-it), Bonn, Germany; g Institute of Global Health, Faculty of Medicine, University of Geneva, Geneva, Switzerland and Swiss School of Public Health, Zürich, Switzerland; h Department of Mathematics, University of Manitoba, Winnipeg, Manitoba, Canada; i MRC Centre for Global Infectious Disease Analysis, School of Public Health, Imperial College London, London, UK; j Centre for Mathematical Modelling of Infectious Diseases, Department of Infectious Disease Epidemiology, London School of Hygiene & Tropical Medicine, London, UK; k National Center for Immunization and Respiratory Diseases (NCIRD), US Centers for Disease Control and Prevention (CDC), Atlanta, GA, USA; l Departement of Computational Biology, Departement of Global Health, Institut Pasteur, Paris, France; m Institut de Physique des Deux Infinis de Lyon (IP2I), UMR5822, IN2P3/CNRS, Université Claude Bernard Lyon 1, Villeurbanne, France; n Mathematical Modelling of Infectious Diseases Unit, Institut Pasteur, Université Paris Cité, UMR2000 CNRS, Paris, France; o Decision and Bayesian Computation, Institut Pasteur, Université Paris Cité, CNRS UMR 3571, France; p Fraunhofer-Institute for Translational Medicine and Pharmacology, Hamburg, Germany; q Institute of Global Health, Faculty of Medicine, University of Geneva, Switzerland; r Mathematics & Statistics Department, College of Engineering and Physical Sciences, University of Guelph, Guelph, Ontario, Canada; s Impact Healthcare, Paris, France; t Respiratory Disease AI Laboratory on Epidemic Intelligence and Medical Big Data Instrument Applications, Department of Engineering Science, Faculty of Innovation Engineering, Macau University of Science and Technology, Taipa, Macau, China; u Africa-Canada Artificial Intelligence and Data Innovation Consortium (ACADIC), Global South Artificial Intelligence for Pandemic and Epidemic Preparedness and Response Network (AI4PEP), Laboratory for Industrial and Applied Mathematics (LIAM), Department of Mathematics and Statistics, York University, Toronto, Ontario, Canada; v Quinten Health, Paris, France; w Airfinity Ltd, London, UK; x Clinical Pharmacy, Saarland University, Saarbrücken, Germany; y Sanofi Vaccine, Lyon, France; z Sorbonne Université, INSERM, Centre de Recherche Saint-Antoine, Paris, France; aa Kap Code, Paris, France; ab Agent-Based Modelling Laboratory, York University, Toronto, Ontario, Canada; ac European Centre for Disease Prevention and Control (ECDC), Stockholm, Sweden; ad UMR 1018, Team “Anti-infective Evasion and Pharmacoepidemiology”, Université Paris-Saclay, UVSQ, INSERM, France; ae Epidemiology and Modelling of Antibiotic Evasion, Institut Pasteur, Université Paris Cité, Paris, France; af Sorbonne Université, INSERM, Pierre Louis Institute of Epidemiology and Public Health, Paris, France; ag Lifen, Paris, France; ah Institute for Computational Biomedicine, RWTH Aachen University, Aachen, Germany; ai Bordeaux University, Department of Public Health, Inserm UMR 1219 Bordeaux Population Health Research Center, Inria SISTM, Bordeaux, France; aj BlueDot, Toronto, Ontario, Canada; ak Fogarty International Center, National Institutes of Health, Bethesda, MD, USA; al York Emergency Mitigation, Engagement, Response, and Governance Institute, Laboratory for Industrial and Applied Mathematics, York University, Toronto, Ontario, Canada; am EHESP, Université de Rennes, CNRS, IEP Rennes, Arènes - UMR 6051, RSMS – Inserm U 1309, Rennes, France

**Keywords:** Modelling, Covid-19, Infectious diseases, Pandemic preparedness, Workshop

## Abstract

In July 2023, the Center of Excellence in Respiratory Pathogens organized a two-day workshop on infectious diseases modelling and the lessons learnt from the Covid-19 pandemic. This report summarizes the rich discussions that occurred during the workshop.

The workshop participants discussed multisource data integration and highlighted the benefits of combining traditional surveillance with more novel data sources like mobility data, social media, and wastewater monitoring. Significant advancements were noted in the development of predictive models, with examples from various countries showcasing the use of machine learning and artificial intelligence in detecting and monitoring disease trends. The role of open collaboration between various stakeholders in modelling was stressed, advocating for the continuation of such partnerships beyond the pandemic. A major gap identified was the absence of a common international framework for data sharing, which is crucial for global pandemic preparedness.

Overall, the workshop underscored the need for robust, adaptable modelling frameworks and the integration of different data sources and collaboration across sectors, as key elements in enhancing future pandemic response and preparedness.

## 1 Introduction

The coronavirus disease 2019 (Covid-19) pandemic brought the field of mathematical modelling into the spotlight, as experts around the world rushed to analyze data for insights into transmission dynamics, disease severity, and potential epidemic trajectories. Once reserved to an audience of specialists, modelling has now become a topic of mainstream conversation. The pandemic made terms like ‘reproduction number (R0)’ and ‘epidemic curve’ familiar to the public, a shift that underscores the increased relevance and accessibility of mathematical modelling (Metcalf et al., 2020). Now, over three years into this global public health crisis, the CERP (Center of Excellence in Respiratory Pathogens) with the support of the AIOLOS (Artificial Intelligence Tools for Outbreak Detection and Response) Consortium, organized a workshop in Lyon, France, on 12th-13th July 2023 aimed at dissecting and learning from the numerous lessons that Covid-19 has conveyed on disease surveillance, mathematical modelling and pandemic preparedness. The workshop brought together mathematical modellers who designed and deployed models used by governments and communities across the world.

Throughout this workshop, numerous accomplishments in the fight against Covid-19 were showcased, celebrating the remarkable progress made. Yet, it is imperative to maintain a balanced view, acknowledging both the strengths and shortcomings of mathematical modelling. Notably, there have been instances of forecasting errors, even in the short-to medium-term projections, and shortcomings in surveillance efforts.

Although the pandemic has highlighted the resilience and strengths of global health systems, it has also emphasized significant gaps and opportunities for improvement. This has spurred countries to develop new pandemic preparedness capabilities, better equipping them to tackle pandemic challenges. These new capacities, fortified by the lessons learned from Covid-19, lay a crucial foundation for enhancing our future pandemic preparedness and response.

The workshop was organized in 6 session, Table 1.

**Table 1:** Workshop sessions.

Overview of different types of data
Lessons learnt from countries
Use case of early warning systems
Innovative approaches to new challenges
Short-term forecasting
Scenario planning and analysis

## 2 Overview of different types of data

In the first session of the workshop, the French-German collaborative project, AIOLOS, which aims to deliver a multi-source platform based on artificial intelligence (AI) and predictive modelling to enhance the early detection and monitoring for respiratory pathogens epidemics and pandemics, was presented. This project co-funded by the French and German authorities involves a consortium of six partners from both countries with complementary expertise: Sanofi, Fraunhofer, CompuGroup Medical, Quinten Health, Umlaut, and Impact Healthcare. AIOLOS collaborates with various public and private stakeholders for data access and technical support.

The consortium arose from the need for real-time surveillance and scenario planning solutions, not only in response to the Covid-19 pandemic but also for management of future epidemics. AIOLOS is structured around a three-step approach: firstly, issue alerts for early detection of new epidemics; subsequently, monitor the progression and impact of these epidemics and; finally, advise appropriate response measures. The technical aspect of this approach involves the real-time integration of data from various sources, including traditional and non-traditional ones like air traffic, mobility, pharmacy consumption, wastewater monitoring, and social media. The analytical component leverages AI and predictive modelling, including natural language processing (NLP) analysis of social media, short-term forecasting, and mid-to-long-term scenario planning. In April 2023, AIOLOS successfully delivered a first minimum viable product (MVP) to validate the models and demonstrate its data integration capabilities using SARS-CoV-2 historical data. This MVP is being will be expanded to cover real-time respiratory virus circulation during the winter 2023–24.

During the session, representatives from five industry partners outlined their diverse data collection and methods experience to support monitoring and prediction of infectious diseases’ outbreaks.

Specializing in wastewater epidemiology, Obepine illustrated how measuring viral concentration can serve as an early indicator of disease prevalence. In April 2020, they reported SARS-CoV-2 concentrations from wastewater treatment facilities around Paris and showed that the first lockdown had a dramatic effect on the virus's circulation; a critical insight at a time when France's clinical testing was not yet comprehensive. From March 2020 to April 2022, their methodology, involving twice-weekly sampling from 150 to 200 wastewater plants, allowed for the identification of viral concentration trends and variant circulation over time. Despite data variability, their mathematical models yielded epidemiological curves that correlated closely with incidence rates measured on individual samples and, in certain cases, anticipated trends up to a week in advance (Cluzel et al., 2022; Wurtzer et al., 2020). Currently, Obepine is broadening their scope to encompass other pathogens and expanding their technological and geographical reach. The SISP&Eau project (https://anrs.fr/fr/actualites/actualites/france-2030-11-projets-laureats-finances-pour-mieux-comprendre-et-se-preparer-a-repondre-aux-maladies-infectieuses-emergentes-pour-pres-de-22-millions-deuros/), supported by PEPR-MIE (Priority Research Programs and Equipment/Emerging Infectious Diseases), will soon be producing a model simultaneously combining data from wastewater analysis and sentinel network data, to assist with early detection of acute respiratory infections.

Kap Code addressed the utility of social media data, demonstrating how AI and NLP can extract patient-generated content into medical ontologies. This digital discourse provides a wealth of data types, including demographics, geolocation, visual, text, social network and user engagement metrics. Kap Code's operations are threefold: observe, by conducting observational studies; engaging patients for clinical trials; and monitor and alert, by implementing monitoring systems for pharmacovigilance and early outbreak detection. Nevertheless, they highlighted significant challenges such as the limitation to public data, platform-specific access rules, and biases intrinsic to social media, including the population that will be investigated and the predominance of negative sentiment.

Focused on the digitization and integration of health data, Lifen presented their work in supporting the healthcare ecosystem. Lifen facilitates hospitals and independent physicians in the digital transformation and organization of health data. By providing a secure communication infrastructure, they ensure that health data exchange remains confidential and efficient. Lifen is advancing a product, ‘Lifen Cohorts’, that capitalizes on their optical character recognition (OCR) and AI capabilities to systematize medical report analysis for robust multicentric patient cohort creation. In collaboration with medical research teams, Lifen has established a framework to manage the regulatory processes crucial for maintaining data integrity and compliance. They start with the automatic importation of data through their previously established systems, ensuring patient anonymity through pseudonymization, then transition to semi-automatic procedures that handle patient eligibility, consent, and the subsequent extraction and exportation of data. This solution allows construction of large patient cohorts using their network of hospitals, thereby leveraging a wealth of detailed information. Every document generated within the hospital setting is a potential data point for the cohort, available for analysis in real-time, or can be used to conduct retrospective or prospective studies. This facilitates the assembly of dynamic cohorts that can evolve with ongoing data collection. Lifen is currently exploring the retrieval of epidemiological data pertinent to infectious diseases. This includes the possibility of flagging specific disease patterns and pinpointing the entry of patients into emergency services, which could be used as early outbreak detection and the monitoring of disease progression.

BlueDot and Airfinity each demonstrated a comprehensive and proactive approach to infectious disease intelligence, leveraging advanced technologies and multidisciplinary expertise to monitor, predict, and simulate disease spread at the population level on a global scale. Both companies function at the intersection of public health and data science, working with governments and the private sector to inform and shape health policy and interventions. They have vast databases of historical health data, which are updated with current inputs from a wide array of sources, both traditional and non-traditional. Their approaches are marked by rigorous data curation and validation, with experts evaluating the relevance and quality of the information. Their proprietary platforms serve as the backbone for generating insights into disease dynamics that are disseminated via user-friendly interfaces such as Application Programming Interfaces (APIs) and dashboards.

BlueDot has supported proactive public health policy and private sector interventions with their approach to infectious disease intelligence that leverages AI, data science, and human expertise. They use a comprehensive database of traditional and novel data sources to detect early signals of global disease activity and assess potential risk of spread and disruption. BlueDot's analytical capacity was particularly evident during the Covid-19 pandemic. They used their global disease surveillance engine for early warning of the emerging pandemic and provided almost daily forecasts on potential virus spread using air travel data, which was particularly noteworthy given the disruption to historical global travel patterns (Fauver et al., 2020). This included publishing the world's first peer reviewed paper on Covid-19 in January 2020 that accurately identified over 10 of the first cities to receive infected travelers from Wuhan, China (Bogoch et al., 2020). Their insights encompass global epidemiological visualizations and country-specific analyses, including updates on viral variants and vaccine impact assessments. For diseases like influenza, BlueDot offers information about the timing of season onset and progression, which can help public health officials and healthcare providers prepare for and manage seasonal outbreaks.

Airfinity extends its forecasting capabilities to a one-month timeframe while also simulating longer-term scenarios that incorporate possible interventions or policy changes. Their portfolio covers a wide spectrum of bio risks associated with more than 150 diseases and probes deep into the epidemiology of many infectious diseases, including targeted attention to Covid-19, influenza, and respiratory syncytial virus (RSV). Airfinity's approach also encompasses a scientific and commercial analysis, aiming to provide stakeholders with a comprehensive understanding of the latest developments and potential market impacts.

In summary, these diverse data sources and methodologies underscore the multifaceted approach necessary for modern infectious disease surveillance and prediction. Emphasizing the importance of integrating various data streams (including ones generated by private sector) and analytical techniques to enhance public health responses.

## 3 Lessons learnt from countries

The second session of the workshop featured an insightful panel discussion with experts from various countries, including France, Germany, Switzerland, the United Kingdom (UK), the United States (US), Canada, China, and the European Center for Disease Prevention and Control (ECDC). The dialogue revolved around two pivotal questions regarding the Covid-19 pandemic:

1. The critical types of epidemiological information utilized during the Covid-19 pandemic.
2. The transformative changes since the pandemic and the lessons that can be leveraged for future outbreaks.

The discussion opened with reflections on the varying importance of data types throughout different stages of the pandemic, highlighting that needs shifted as the pandemic unfolded. Initially, sparse international case data provided the first indication of interhuman transmission, underlining the importance of early spread information. As the pandemic progressed, robust severity estimates became vital, but it was challenging to obtain accurate data. For the subsequent waves, the debate intensified over the most effective intervention strategies. Upon the deployment of vaccines, estimates of vaccine effectiveness played a key role. Models helped communicate to policymakers and the public that vaccines were part of a broader response strategy. The varying data needs underscored the requirement for a robust mix of data collection methods, combining traditional surveillance, including syndromic data, testing, death certificates, and other more innovative approaches.

Human behavior emerged as a significant factor influencing both transmission rates and vaccine uptake, highlighting a gap in real-time behavioral data. The integration of behavioral data into epidemiological models was a significant advancement. The ECDC, for instance, combined expertise in behavior analysis with epidemiology, forging a comprehensive interdisciplinary approach that elucidated contact patterns and vulnerabilities.

While many countries had access to digital contact tracing technologies, the opportunity to leverage these tools effectively was not fully realized. The challenges lay in data privacy issues, and the in the optimization and data collection, where potential insights were lost due to technological and operational shortcomings. The UK's experience, however, stood out as a positive example.

The granularity of geographical data also emerged as a critical theme. The UK's experience with regional-level predictions, and France's success in utilizing regional forecasts for lockdown decision making and cross-regional hospital patient transfers during lockdowns, highlighted the value of localized data for real-time decision-making. Yet, in federated countries like Switzerland, Germany and the US, where each state has a lot of independence and there are many different stakeholders, differing state-level responses led to complexities in data standardization and comparability.

Throughout the Covid-19 pandemic, interest was not only on the acquisition of data but also on the strategic framework within which the data were used. The panelists mentioned that gaps in various countries' surveillance strategies were not due to a lack of data but rather due to the absence of a cohesive rationale guiding surveillance objectives. Critical reflection on the central questions that surveillance aimed to address was also often missing. Robust system designs are imperative to ensure resilience and adaptability. However, the possession of high-quality data does not directly translate into optimal decision-making. Decision-makers can be influenced by various factors beyond data, including personal judgment and political considerations. Acknowledging the human element in public health responses underscores that while data are crucial, it is not the sole determinant of a successful strategy.

For improved future responses, it is essential to cultivate strong relationships between the public health community, policymakers, statisticians and the data modelling community. This requires a commitment to enhance the understanding of mechanistic modelling among policymakers, enabling them to grasp how models inform potential responses. Investment in this area has been evident, with policymakers showing an increased willingness to comprehend and apply modelling tools. The demand for scientists in health ministries and other governmental bodies has also become apparent, advocating for a science-based approach to policy-making. The pandemic served to emphasize the importance of epidemiological modelling in public health decision-making. Sustaining this momentum of ‘model literacy’ and engagement is vital for preparedness in future pandemics. Scientific journalism was a bridge between complex scientific assessments and the public, demonstrating an improved capability to distil scientific findings into understandable narratives. Similarly, it is the modellers responsibility to ensure that their outputs are question-driven and transparently presented.

The pandemic also catalyzed unprecedented cooperation and knowledge exchange among modelling groups worldwide, particularly in the US and Europe with the creation of modelling hubs. Additionally, in France there was a shift in the dynamics between modelling groups, which historically, had little to no interaction, but during the pandemic developed formal channels of communication and collaboration.

Nonetheless, data issues persisted in the form of achieving data FAIRness—ensuring data are Findable, Accessible, Interoperable, and Reusable. Despite the availability of vast datasets on vaccines, cases, and hospitalizations, challenges remain due to regulatory limitations and the difficulty of linking datasets. Going forward, enhancing technological capabilities to interconnect various data sources is crucial for holistic surveillance and public health strategy formulation.

The experts called for more cooperative and combined approaches to data management, with a view to avoid duplication of efforts in data collection and infrastructures and toward breaking down silos. They also highlighted the critical nature of data ownership and cost, with private companies increasingly gatekeeping essential research and development data, from one side to preserve intellectual property and public sector and academics gatekeeping some epidemiological and patients’ data based on visibility and funding opportunities. This led to discussions about frameworks for equitable data sharing and access, drawing parallels with the for genetic resources (https://www.cbd.int/abs/doc/protocol/nagoya-protocol-en.pdf). The need for equitable access to data and resources, especially for countries unable to afford them, was underscored as a priority for international health.

In anticipation of future pandemics, panelists underscored the importance of health economics studies and the need for infrastructure to conduct real-time, ethically sound trials. The concept that ‘seasonal preparedness is pandemic preparedness’ was advocated, stressing the continuity of analytic practices outside of crisis periods, notably by modelling the dynamics of seasonal respiratory viruses to ensure sustainability and readiness. The momentum gained in data analysis and modelling needs to be maintained, ensuring that lessons from Covid-19 are applied to prepare for and mitigate the impact of future pandemics.

## 4 Use case of early warning systems

During the next session four presentations discussed different types of models, and data sources as tools for preparedness for future pandemics.

### 4.1 Variant-driven early warning system via unsupervised machine learning analysis of spike protein mutations for SARS-CoV-2

Giacomo Cacciapaglia presented on early detection of Covid-19 variants, and the importance of tracking the emergence of new variants of epidemiological significance. To identify, classify and track relevant variants of SARS-CoV-2, an unsupervised machine learning model was developed, that doesn't rely on statistical models or prior knowledge about the virus's variants (De Hoffer et al., 2022). The algorithm used the Levenshtein measure, a metric that measures the distance between protein sequences based on the number of amino acid substitutions. Using sequences from the viral spike protein through hierarchical clustering, similar sequences grouped together, entirely free from bias. To perform this type of analysis, typically 100 sequences were required per time unit. Each time unit may include more clusters and, within each cluster, the dominant variant is the most frequent in terms of identical sequences over the total number of sequences in the cluster. Next, an algorithm was developed to connect clusters that appear in consecutive time units, forming chains of clusters sharing the same dominant spike variant. Empirically it was determined that chains persisting for more than three-time units indicated emerging variants of epidemiological significance.

The system effectively serves as an early warning mechanism for emerging variants, activating once the associated cluster reaches 1% of the time-binned sequence data. To validate this approach, UK data from the platform Global Initiative on Sharing All Influenza Data (GISAID) spanning 2020 and 2021 was used, and successfully replicated the waves from the original Wuhan strain to the Alpha and Delta variants. This pattern underscores the strong correlation between variant emergence and the temporal multi-wave pattern of the pandemic. Drawing inspiration from theoretical physics, specifically the concept of ‘fixed-point’ dynamics, this model provides insights into understanding the dynamics of Covid-19 variants (Cacciapaglia et al., 2022; Della Morte et al., 2020).

This exploratory analysis is being expanded to test the robustness of this approach especially in reducing the number of sequences needed and looking at other countries like France and Germany.

### 4.2 Early warning system and prediction model for Covid-19 in China

Chitin Hon presented on the work from China, with a research framework constructed around three primary data sources: epidemic data sourced from sentinel sites, hospitals, the national Centers for Disease Control and Prevention (CDC), and testing institutions; pathogen data including genomic sequences; and public opinion data gathered from social media. The forecasting exercise was organized into three main phases: data gathering, trend analysis, and the development of an early warning strategy.

Given the evolving nature of the pandemic in China, the objectives of the prediction models shifted across different phases. During the Covid-Zero policy period (January 2020 to December 2022), the models aimed to predict key metrics such as the peak time and intensity of outbreaks, the total number of infections, and the duration of waves, as well as mortality rates and the demand for critical care resources. Subsequently, after the relaxation of the Covid-Zero policy in 2023, the models focused on predicting the timing of peak infection rates and the occurrence of future waves, as well as continued forecasts for mortality and healthcare resource demand.

Two distinct modelling approaches were employed during these two periods. In the Covid-Zero phase, before the optimization of epidemic control measures, the research team employed the Adaptive Fourier Decomposition (AFD) method, an enhanced version of Empirical Mode Decomposition. AFD was combined with Long Short-Term Memory (LSTM) modelling. This approach was effective in decomposing historical trends and allowed the team to extract critical information from 108 epidemic waves with infection scales exceeding 300 people since the onset of the Covid-19 pandemic. By analyzing these trends, the researchers discovered strong correlations between total infections and AFD trend peak value, as well as total infection and peak value. Leveraging these historical data trends, the models could predict 80% of the total number of infections and the end dates of the initial waves. In some Chinese cities, predictive deviations were found to be less than 5%.

In the subsequent phase, after the relaxation of control measures, a modified Susceptible-Exposed-Infected-Removed (SEIR) transmission dynamics model was employed to forecast the epidemic trend. This modified SEIR model incorporated a parameter accounting for the protection rate against re-infection among the previously infected population, which decreased over time. While this SEIR model provided insights into the epidemic trend, AFD and LSTM models were still necessary to predict seasonality. This combined approach proved effective in predicting the peak and scale of SARS-CoV-2 outbreaks following the relaxation of control measures, including forecasting the April and May 2023 waves at the end of 2022.

One notable data source utilized in this research was the “Pandemic Forecast and Warning WeChat Mini Program” developed as part of a national project aimed at predicting Covid-19 trends based on big data. An online questionnaire within this platform enabled the random collection of information related to fever-based infectious diseases. The program aggregated infection data from all 31 provinces in China, leveraging the widespread use of WeChat, a Chinese instant messaging social media, to enable public-inclusive early warning and forecasts. Furthermore, a public sentiment surveillance tool was implemented to provide complementary information about the epidemic, in advance of official reports.

### 4.3 Early warning system in Africa

Jude Kong shared the experience of the “Africa-Canada Artificial Intelligence and Data Innovation Consortium” during Covid-19 and how this can be leveraged and capitalized for other respiratory diseases (https://acadic.org/covid-19-dashboards/) (Kong et al., 2023; Stevenson et al., 2021). The consortium, established in 2020 with a focus on fostering knowledge sharing, operates across ten African countries. The consortium's mission centers around addressing the challenge of obtaining disease data from remote and underserved communities that often goes unreported in centralized systems. Their primary goal is to develop proactive tools tailored to inform locally relevant policies. This endeavor is characterized by a collaborative, interdisciplinary approach that places the community at the center of the decision-making process, working in tandem with governments to ensure successful implementation.

During the pandemic, the consortium devised a comprehensive modelling and forecasting strategy (Kong et al., 2023). Their approach revolved around sourcing data from community-led organizations and partnering with telecommunications giants, Orange and MTN, to collect vital community-level information. Once data were amassed, they were processed into long- and short-term memory datasets, serving as the foundation for training a recurrent neural network model capable of predicting disease trends up to 14 days in advance (Stevenson et al., 2021). This predictive model was then cross-referenced with historical outbreak data to establish a value threshold. Whenever the predicted values surpassed this threshold, an alert was triggered, signaling a potential outbreak. This method proved to be effective in monitoring Covid-19 developments across some African countries, emphasizing its community-based, rather than country-specific, approach.

Building upon this success, the consortium expanded its efforts to encompass other diseases. They developed an AI-powered air quality monitoring device, which forecasts air quality in various communities and correlated it with case count of respiratory diseases. Moreover, the consortium designed a comprehensive dashboard that merges data from sources such as Wiki, Google, Facebook, and OneNote. When adapted for Mpox and Covid-19, this tool demonstrated an 80% accuracy rate in detecting and predicting disease trends in different countries. When it comes to Africa, they successfully integrated the dashboard with community health data, and while this area of the early warning framework is still in development it has already provided advanced warnings with a 30-day lead time.

### 4.4 Early detection of respiratory viruses applied to Germany

In the fourth presentation, Holger Frohlich summarized their results and motivation to use social media as an additional complement to traditional surveillance data for early detection of Covid-19 in Germany. Despite the inherent challenges and biases associated with such data, and potential cultural variations in social media behavior across countries, Google searches and Twitter can be valuable data sources.

The methodology involved the construction of a corpus of disease symptoms derived from automated literature mining of articles discussing Covid-19 symptoms. Subsequently, terms that were disproportionately mentioned compared with chance were identified. A similar process was applied to Google Trends and Twitter data, and a list of top terms was compiled. The team performed trend analyses on these digital traces and compared them with surveillance data to determine correlations and forecast trends. To account for seasonal trends, the LOESS method was used (Cleveland et al., 1990) and due to pandemic events exhibiting exponential behavior within a relatively short time frame, log-linear regression models were developed. These models were fitted over sliding windows and evaluated for statistical significance of the slopes. This process was repeated for all symptoms and combined into a joint indicator using the harmonic p-value method. The resulting indicator was then compared to surveillance data. The findings indicated that social media data provided an early alert for uptrends, often preceding the official reporting of cases in Germany by about a week. Analysis of time lags revealed that, on average, two weeks in advance, uptrends and downtrends in surveillance data could be predicted (Wang et al., 2023).

To enhance predictive capabilities, the team developed a forecasting model using LSTM, trained as well on a sliding window approach to make 14-day ahead predictions for confirmed cases and hospitalizations. Comparative analysis with a random forest model favored LSTM. Performance metrics, including sensitivity, precision, and F1 score, suggested that the approach was effective, even for downtrends. Model interpretability was explored using the Shapley Additive Explanations approach (Lundberg & Lee, 2016), identifying influential terms reflecting common Covid-19 symptoms reported by the public.

## 5 Innovative approaches to new challenges

The following session really showed on how during times of crisis innovation and creativity can expand. The Covid-19 response ignited numerous innovations, maintaining momentum and engagement will make it possible to build on the scientific gains and lessons from Covid-19.

### 5.1 Hybrid modelling by knowledge integrated machine learning in infection dynamics - lessons learned from the SARS-CoV-2 pandemic

Andreas Schuppert, recounted his experience in assisting intensive care colleagues with predictions about intensive care unit (ICU) capacities in Germany during the pandemic's early waves. He noted the challenges faced due to a lack of data and understanding at that time. He emphasized the need for an approach that integrates both traditional knowledge and machine learning, termed ‘hybrid modelling’. This method combines a priori knowledge, such as physics-inspired neural networks and transfer learning, with machine learning to enhance prediction accuracy. He highlighted the benefits of this approach, like needing significantly less data than traditional models and its ability to extrapolate into sparsely covered data spaces. He focused on the development and application of ‘structured hybrid modelling’. This concept, originating in chemical engineering and extensively influenced by mathematics, involves decomposing a black box model into a network of functionalities and interactions. Each node in this network represents a specific functionality, which can be modeled through various methods, including physics-based models and neural networks.

Andreas shared insights from applying this methodology during the pandemic. For example, integrating a Susceptible-Infected-Recovered (SIR) model with a LSTM network helped analyze the infection dynamics of the first and second waves in Germany. This approach allowed for the examination of various factors like population density and age distribution and their impact on the pandemic's trajectory. Furthermore, he discussed the challenges of using mean field models like SIR, which often assume exponential dynamics, and how their application to German data revealed variations in infection dynamics across different regions and times.

This presentation stressed the importance of developing more efficient tools that integrate machine learning and traditional mechanisms for future pandemic preparedness and response. The difficulties posed by missing data and incomplete understanding of infection mechanisms, which hamper reliable predictions and optimal response strategies were also acknowledged.

### 5.2 Accounting for the co-circulation of multiple pathogens

Lulla Opatowski presented integrated methods that account for the co-circulation of different pathogens. She emphasized that numerous microorganisms, including pathogenic bacteria and respiratory viruses, coexist in the respiratory tract and possibly interacting within the human population. Most mathematical models tend, however, to overlook these intricate interactions and the co-circulation of pathogens which may play a role in the global dynamics.

An overview was presented on how to incorporate interactions between viruses and bacteria, as well as viruses with other viruses, into transmission models (Arduin et al., 2018; Domenech De Cellès et al., 2019; Opatowski et al., 2018). This approach, when confronted to data, can help untangle the various factors contributing to disease incidence and determine and describe the role of viruses in the development of bacterial infections. While most studies have primarily focused on influenza interactions, recent reports have looked at SARS-CoV-2's: epidemiological studies indicate that prior influenza virus infection may increase the risk of SARS-CoV-2 infection and disease severity; animal studies suggest that co-infection with influenza virus and SARS-CoV-2 results in more severe disease than mono-infection with either virus (Wong et al., 2023). An interesting example was shown of how a multi-pathogen susceptible-exposed-infectious-recovered transmission model, accounting for the co-circulation of SARS-CoV-2 and another respiratory virus (e.g. rhinovirus), can analyze the impact and possible bias of such circulation on surveillance indicators of SARS-CoV-2 (especially the positivity rate in viral surveillance). Correction of SARS-CoV-2 positivity rate can, however, be achieved by conducting multiplex PCR on a small number of samples (Kovacevic et al., 2022).

To explore the patterns of co-detection over time, in an ongoing analysis of surveillance data from respiratory hospital admissions in Spain over a decade, multi-virus co-detections are being analyzed by incorporating different interaction mechanisms, such as modified susceptibility, hospitalization risk, residual positivity, and infectiousness.

The talk also highlighted the interactions between influenza and bacteria (pneumococcus), stressing age-dependent dynamics and suggesting that interventions targeting viruses could have a substantial impact on bacterial epidemiology. Controlling one virus could significantly influence the dynamics of other circulating pathogens, which can have public health implications. Incorporating these interactions into models enables a more accurate evaluation and anticipation of the timing, burden, and global impact of public health interventions. However, it is necessary to acknowledge the challenges involved, particularly the need for better understanding and quantifying the strength of these interactions at the individual and the population levels. Furthermore, complex models that consider multiple pathogens together pose a significant challenge of parameterization. It was also mentioned that current surveillance data are very limited on co-infections data, generally providing very little ecological information, which constraints in-depth investigations into these interactions. For a more comprehensive understanding, multi-pathogen surveillance at a larger scale and individual-level data are necessary.

### 5.3 A framework for behavior quantification vis-a-vis adoption of public policy measures - lessons from nonpharmaceutical measures during Covid-19 in Ontario

Monica Cojocaru presented on the complexities of assessing behavior against the backdrop of public health directives, drawing insights from the deployment of nonpharmaceutical interventions (NPIs) during the Covid-19 pandemic in Ontario, Canada. The goal was to detail the patterns of disease spread within the province. They sought to understand the public's perception of the risk of contracting the virus and the unease brought about by NPIs, evaluating how these perceptions influenced their acceptance and adherence to such measures. To quantify these attitudes and their subsequent behaviors, the research team formulated a decision model capturing the decision-making processes of individuals, taking into account the information available to them and its impact on viral spread. The aim was to discern the actual level of compliance with NPIs across Ontario's 34 diverse public health units (PHUs).

The team applied multilinear regression techniques to estimate the perceived risk of infection and the discomfort associated with NPIs on a monthly basis for each PHU. Their findings revealed that while population density influenced perceived infection risk, it had no bearing on the personal discomfort of NPIs like mask-wearing. This highlighted that the sense of restriction from NPIs is a personal sentiment, seemingly unaffected by the density of population in the individual's environment. Further analysis considering factors such as age, income, education level, and local climate, showed that personal discomfort was an isolated variable, not significantly linked to any of these factors. In contrast, infection risk perception was found to be directly proportional to the median age and inversely related to median income (Fields et al., 2021).

Game theory was then incorporated to predict the likelihood of an individual adhering to NPIs within each PHU. Each ‘average’ person was assumed to weigh the epidemiological and policy information daily against their personal discomfort to decide whether to comply with NPIs or increased risk of infection. This allowed the team to map out expected compliance with NPIs week by week from March to December 2020. The outcomes of these compliance predictions were then plotted against the trajectory of the pandemic in Ontario. Utilizing a SEIRL (Susceptible, Exposed, Infectious, Recovered, and Isolated)-type model, fitted to early-stage pandemic data, they were able to deduce that adherence to NPIs mitigated transmission rates. They calculated the R0 for each PHU and the overall effective R0 for the province, which helped create a timeline of the initial spread of the infection based on the initial R0. The model's predictions on NPI compliance were then matched against the actual case data from Ontario, showing a strong correlation.

A potential limitation of the study was the likelihood of spatial dependency due to interaction between populations across PHUs and spatial regression models could provide a more appropriate fit for this problem.

### 5.4 NLP method on social media data

Kap Code presented on an advanced application of their proprietary social network analysis, leveraging AI and text mining techniques to dissect the discourse of the Covid-19 pandemic across social media platforms. Their aim was to identify and track self-reported symptoms of Covid-19 as articulated by users in Chinese, English, and French, scrutinizing the progression of these discussions and noting linguistic disparities. To achieve this, they harvested a corpus of Covid-19-centric messages during the initial lockdown, curating a symptoms list from scholarly sources and the MedDRA's comprehensive medical terminology. Utilizing their specialized fuzzy matching algorithm, they monitored and contrasted the symptom prevalence as reported in each of the three languages. Their results showed that psychological symptoms were the most discussed symptoms on the three languages, while fatigue and respiratory symptoms were the most prevalent physical symptoms mentioned. Notably, misinformation threads were detected, irrespective of language, from the beginning of the first lockdown.

Focusing specifically on Twitter data, Kap Code sought to assess the reporting frequency of anosmia and ageusia, symptoms later recognized as Covid-19 indicators, to determine if these associations could have been flagged earlier on the platform. By mining early pandemic-era tweets and employing their fuzzy algorithm, they delineated the trajectory of public discourse around these symptoms and gauged the impact of media coverage on public health literacy. Although an early association via Twitter data alone was not possible, the platform's quick uptake of relevant news content was evident.

In a comparative analysis of Chinese and French social media during the lockdown, Kap Code applied their topic modelling to messages from distinct phases: pre-lockdown, early lockdown, and late lockdown. Through this, they categorized discussions in both countries into four broad themes: epidemic news and daily life, scientific information, public measures, and solidarity/encouragement. The analysis revealed a more scientific content in Chinese posts, a more critical stance towards public measures in French discourse, and a common thread of misinformation in both languages.

Lastly, within the AIOLOS project, Kap Code developed an algorithm aimed at the early detection of respiratory syndromes on social media in France, a tool with the potential to anticipate seasonal peaks in patient numbers. Their method involved a three-stage extraction and filtration process to single out medical terms linked to respiratory illnesses, followed by an analytical time-series method allowing for both backward and forward-looking weekly projections.

### 5.5 NLP for epidemiology: determining circumstances of Covid-19 transmission from ComCor free text data

Gaston Bizel-Bizellot presented on team's research on the use of NLP to investigate patterns of SARS-CoV-2 transmission from the text responses of the large-scale French ComCor study, which spanned from October 2020 to February 2022 (Charmet et al., 2021; Galmiche et al., 2021; Grant et al., 2022). This nationwide case-control study, provided a unique opportunity to test the application of NLP methods on survey data. Specifically, they assessed whether NLP methods could apply to surveys and expand the typical survey approach of closed-ended questions to more open-ended questions, with free text responses. They employed CamemBERT, a model based on the BERT architecture but pre-trained specifically on a comprehensive French corpus, which is designed to capture semantic and syntactic relationships within sentences (Devlin et al., 2019; Martin et al., 2020). The ComCor survey's structure, with its mix of open and closed-ended questions, provided a rich dataset for this approach. The team started with a validation phase, using the survey's paired questions—linking the open-ended responses to their closed-ended counterparts. After training on a segment of the ComCor dataset, they evaluated a classification model that provided categorical probabilities. The model achieved a prediction accuracy of 75%, indicating that the text responses held significant informational value that CamemBERT was effectively able to extract.

In the next step, they proceeded with an unsupervised approach, disregarding the closed-ended questions to focus purely on the text data. They applied the BERTopic framework for data visualization, which clusters individual responses into topics (Grootendorst, 2022). This framework revealed new, organically formed clusters. This unsupervised clustering not only recovered the predefined categories but also surfaced additional categories and allowed for a deeper understanding of the varied circumstances of viral transmission.

### 5.6 From multi-armed bandits to decisions in complex varying environments

Alex Barbier--Chebbah presented on the application of multi-armed bandit systems in the context of pandemic decision-making, highlighting their potential for optimizing the allocation of limited resources. The multi-armed bandit problem represents a fundamental challenge in reinforcement learning, showcasing the tension between exploring new strategies and exploiting known ones to maximize expected gains. This issue becomes particularly salient when each choice's outcomes are only partially known and may unfold over time as resources are invested. In practical terms, Alex suggested using multi-armed bandit algorithms to manage ensemble stacking of pandemic predictors. Such predictors attempt to model the dynamics of the pandemic but are often hampered by noisy data and inevitable delays in information availability. The question then arises: which predictor should be relied upon? Rather than averaging the output of all predictors, he proposed a dynamic weighting system that could more effectively adapt to new and changing policy contexts as well as to high variability in data. This could be done by modelling each predictor of the ensemble as an ‘arm’, with dynamically adjusted weights over time to minimize its expected losses and manage the predictions. Instead of committing to a static ensemble approach, this system would allow for a more flexible aggregation of predictions requiring less data to be trained, and seamlessly handling data revisions or the integration of new predictors during pandemic. Additionally, it was pointed out the feasibility of deploying a large number of these systems, focusing not on testing every single arm but on identifying and leveraging the sufficiently promising ones, calling for future investigations in drug testing or resource allocation for epidemic monitoring.

Such adaptive systems, could offer a way to engage with an evolving pandemic landscape, allowing for the real-time updating of beliefs and strategic allocation of effort to yield the most informative and beneficial outcomes.

### 5.7 US Covid-19 scenario modelling hub

Cecile Viboud presented on the US Covid-19 modelling hub, which was launched in December 2020 parallel to the release of the first vaccines in the country. This collaborative effort integrates a multitude of academic teams and US agency partners, focusing on projections spanning 3–24 months. The hub aims to provide actionable projections to inform public health decision-making and to advance the science of disease forecasting.

The initiative facilitates the combination of various models through an ensemble approach, wherein each model is required to produce probabilistic projections within the scope of numerous considered scenarios employing epidemic drivers. To date, the hub has completed 16 rounds of projections. Central to the hub is defining the scenarios, each normally incorporating two epidemic drivers among vaccination rates, NPIs, variant emergence, and immunity waning. Vaccination strategies have been a persistent focus, with 11 out of the 16 rounds including this factor. More recently, the emergence of variants and the implications of waning of immunity have been highlighted. The June 26, 2023, release was termed the ‘mega round’ due to its expansive analysis of six scenarios and projections extending two years into the future. The scenarios considered variables such as rates of immune escape and different booster vaccination strategies. Despite the varying scenarios, hospitalization levels are expected to remain consistent with the previous year's data, and annual deaths are projected to range from 45,000 to 97,000. A broad vaccine recommendation was projected to reduce hospitalizations and deaths by 8–13%, which translates to significant figures when applied to the US population over two years—around 200,000 hospitalizations and 17,000 deaths could potentially be averted.

Doing a deep dive on the performance of the projections over the last two years, by analyzing the plausibility of the scenarios and the accuracy of the forecasts using measures like the 95% coverage probability and the weighted interval score, it was recognized the superior performance of ensembled projections over individual models and the importance of maintaining variation between models (Howerton et al., 2023). However, it was also acknowledged a limit to predictability, primarily due to the emergence of new strains, with an effective projection horizon of about 22 weeks. As the virus transitions to an endemic stage this might however change.

## 6 Short-term forecasting

A session on short-term forecasting followed showcasing five countries.

### 6.1 FluSight: real-time forecasting for influenza

The first speaker, Rebecca Borchering (US CDC), began by describing the state of influenza forecasting in the US back in the early 2010s: a large number of forecast models existed, most publishing results months to years after the fact, using diverse forecast targets as well as different evaluation metrics for forecast performance. During the course of a season, it was thus not possible to obtain truly comprehensive, interpretable and real-time views. Into this landscape, CDC launched the “Predict the influenza season challenge” for the 2013–2014 season. Its objectives were to improve the awareness and understanding of influenza forecasting models, to drive the development of forecasting methodology and the use of novel data sources, and in the process to build a community of forecasters collaborating with the CDC (Lutz et al., 2019; McGowan et al., 2019; Reich et al., 2019) (https://www.cdc.gov/flu/weekly/flusight/index.html). The output of this initiative would be used to complement CDC's existing systems for monitoring influenza. FluSight has operated for every influenza season since, with the exception of 2020–2021, when the Covid-19 pandemic severely disrupted the respiratory virus season (Olsen et al., 2021). Similar initiatives were later launched for dengue (2015) (Johansson et al., 2019), Aedes mosquitoes (2019–2020), and West Nile virus neuroinvasive disease (2020, 2022 and 2023).

FluSight has been very successful in making influenza forecasting more timely and standardized, and in community-building among researchers. Moreover, the experience gained and methodology developed from 2013 to 2019 served as the backbone for CDC's Covid-19 forecasting efforts. In turn, Covid-19 induced a key change in FluSight. Up to and including the (truncated) 2019–2020 season, the forecast targets were focused on influenza-like illness (ILI) data (U.S. Outpatient Influenza, 2023), but due in part to similarity in symptoms, this was no longer considered an ideal indicator of influenza burden after the onset of the Covid-19 pandemic. The forecasting target was thus changed to the weekly number of laboratory-confirmed influenza hospital admissions; these data are collected across the US at the facility level and made available through the HHS Protect Public Data Hub (COVID-19 Reported Patient Impact and, 2023) (FluSight Forecast Hub, 2023) (Mathis et al., 2023; COVID-19 Guidance for Hospital Reporting, 2023).

In retrospectively evaluating the performance of contributing teams’ forecasts across each influenza season, some key lessons have been learned and reinforced: the first is that individual forecasts tend to vary in performance within and across seasons, but ensembles have more consistent performance, and on average have been among the highest-performing forecasts. Second, is that forecast performance tends to decrease with increasing time horizon. This is of course not a surprise, and simply reflects the fact that forecasting further into the future is harder. Thirdly, whether from individual models or ensembles, forecasts do not yet reliably predict changes in trends. For example, in the 2022–2023 influenza season, which began unusually early, most models underpredicted influenza hospital admissions in the initial growth phase, but then overpredicted the peak. A major challenge, therefore, is to find ways to improve forecast performance at the inflection points of a season, whether through improvements in the models themselves, through finding novel data sources that can serve as leading indicators of trend changes (e.g. wastewater surveillance), or through improvements in the ensembling of forecasts.

### 6.2 Using ‘forecasts’ to inform policy-making

Marc Baguelin (Imperial College London, UK), started by introducing the UK Scientific Pandemic Influenza Group on Modelling, Operational Subgroup (SPI-M-O). When called during emergencies, SPI-M-O reports to the UK Scientific Advisory Group for Emergencies (SAGE), which then advises the government. It focuses exclusively on epidemiological modelling (with no element of for example economics or behavior), and includes many UK universities. SPI-M-O itself does not make or suggest policy decisions. The importance of recognizing the differences between the concepts of projections, scenarios, central scenario, reasonable worst-case scenario and nowcasts which are often lumped together as “forecasts” was emphasized. Forecasts themselves should be thought of as a set of future outcomes associated with a probability distribution. Projections, in contrast, are an extrapolation of the current situation assuming conditions (in model terms, the parameters) remain constant. Scenarios, meanwhile, are extrapolations conditional on assumptions about changes in the conditions/parameters, such as a ‘reasonable worst-case’ scenario (e.g., assuming poor but still plausible future NPI compliance and vaccine uptake). Finally, there are also nowcasts, which are a probability distribution of the current state (e.g., total daily number of new Covid-19 cases) considering uncertainty in the understanding of the current state (e.g., due to limited testing).

SPI-M-O does not produce forecasts in the true sense, for a couple of reasons. First, future outcomes depend on factors outside the remit of SPI-M-O, (i.e. human behavior, policy decisions and virus biology). Second, forecasts themselves can affect behavior and/or policy, thus making the forecast ‘wrong’. Nevertheless, in practice, even with these disclaimers, SPI-M-O's scenario models - typically for time frames of 8 weeks into the future - have been widely interpreted by the public and media outlets as ‘predictions’. What SPI-M-O produces can be categorized as i) nowcasts (of R0, incidence and prevalence of hospitalization, prevalence of infection), ii) medium-term projections over two to six weeks, iii) scenarios associated with medium-term projections, iv) specially commissioned work to inform specific decisions, with multiple scenarios being explored, and v) other bespoke group modelling work involving at least three distinct groups to develop consensus statements. In the UK, users of the above products have included the cabinet office, the government and parts of the National Health Service, as well as other civil service departments. They have used them for planning and decision-making. Also, consensus statements deriving from these products have been regularly released to the public.

As a case study the SPI-M-O consensus statement on Step 3 of the UK's roadmap for easing of Covid-19 restrictions in the spring of 2021 was also described (Sonabend et al., 2021). He emphasized that an important component of this document was the scrupulous use of cautions about assumptions and things not accounted for, such as changes in behavior, the progression of the ongoing vaccine rollout, and the emergence of new variants. Overall, SPI-M-O's input into the roadmap has been seen as a success story in the integration of science and policy (Sonabend et al., 2021). Most recently (as of the date of this workshop), some restrictions have been re-applied in the UK, and modelling continues to feed into policy.

### 6.3 Forecasting the Covid-19 healthcare demand: the case of France

Simon Cauchemez (Institut Pasteur, France) described the work done by his team in forecasting healthcare demand due to Covid-19. Initially a single mechanistic transmission model was used, calibrated on hospitalization data. As additional types of data became available, the team decided to explore whether inclusion of these data to the modelling framework could increase forecasting power. For the second wave of the pandemic, they developed an ensemble of statistical models, which made use of earlier predictors of SARS-CoV-2 infection (as compared to hospitalization, which is a lagging indicator). The models included autoregressive integrated moving average (ARIMA) models, a generalized additive model (GAM), a distributed lag model, a boosted regression tree model, and a random forest model. Predictors included Google mobility indices, proportion of positive tests, temperature, and humidity, with influential predictors being selected by a forward stepwise procedure. The ensemble forecast was the unweighted mean of the individual models’ forecasts. In contrast to the multi-team FluSight ensemble (see previous presentation), this was an ensemble of multiple models developed by a single team. Model performance, was best for models that used early predictors as inputs. And, similar to FluSight, the ensemble model on average outperformed all of its component models. Model performance was subsequently found to improve on average when using only models that utilized epidemiological inputs, though a later re-analysis showed that incorporating mobility significantly improved performance in some situations. A later development has been a model that exploits the more regular, periodic nature of Covid-19 incidence since the appearance of the Omicron variant; it uses a piecewise-sinusoidal model fit for each new wave, with the previous wave as a (Bayesian) prior.

The speaker ended by pointing out that important datasets for his group's (and many other's) models now are no longer available, notably Google mobility data and key Covid-19 case and hospitalization surveillance data in France.

### 6.4 Modelling and simulation of SARS-CoV-2 infections, hospitalizations and outcome in Germany

Thorsten Lehr (Saarland University, Germany) presented on a compartmental model (Dings et al., 2022), implemented with a stepwise approach, going from infection to hospitalization to outcome, that was applied at different levels: Germany-wide, the federal state (Bundesland, or NUTS -1), the government region (Regierungsbezirk, NUTS-2) and the county (Landkreis, NUTS-3). The objective from the beginning was to make the results publicly available. The model used multiple data sources, but a particularly useful and reliable source was the CGM MetaKIS database, covering about 10% of hospitalized patients in Germany. Forecast performance was found to be better at the intermediate government region resolution than the higher county level resolution; the latter often separates hospitals from parts of their catchment areas. A dashboard interface to the model was made publicly available (https://covid-simulator.com/). At peak usage, the model was being accessed about 200,000 times per day. The model was also applied to Switzerland, Spain, Italy, France, Luxembourg and the US.

The research group's lessons learned were: i) mathematical models are useful tools in a pandemic, ii) data availability and ‘the pandemic moving faster than the science’ are a constant challenge, iii) long-term forecasting is impossible unless one finds a way to predict the behavior of the population and of politicians (i.e., public policy), iv) hospitalizations, ICU occupancy and outcomes are predictable if the incidence of infections is known, v) simulation of ‘what-if’ scenarios is important to assess interventions, and vi) scientific communication to all stakeholders - public, political, media - requires care and forethought.

The speaker ended by talking about SaarCoKids, a project run in multiple Saarland schools and childcare centers after the resumption of the 2020/21 school year. In it, student location and density were measured via radio transmitters, thus allowing detailed characterization of in-school contact networks. Analysis of the data is ongoing, but a preliminary finding is that teachers play a more important in these contact networks than was previously realized.

### 6.5 Short-term forecasting: experience from Canada

Jianhong Wu (York University, Canada) explained that the SARS-CoV-1 outbreak of 2003 gave Canada some valuable experience in creating collaborations between modelling consortia and decision-making bodies. This helped Canada mount a rapid modelling response to Covid-19, with a national modelling task force being established on February 4, 2020, which was incorporated into the Public Health Agency of Canada's Modelling Expert Group for Covid-19 two weeks later. This taskforce advised on the development of Canada's initial escalation strategy of Covid-19 response measures, from early monitoring and international travel advisories, to the closure of all non-essential workplaces on March 24, 2020. In the province of Ontario, the Modelling Consensus Table was formed on March 25, 2020, as part of the Ontario Science Advisory Table. Early Ontario near-casting work by his research group used simple ‘model-free’ linear regression, informed by data from the further-progressed pandemic in Italy, to project case incidence through the end of March. In early April 2020, non-linearity made this approach no longer working well, and his group switched to using a dynamic transmission model to make forecasts.

A key early question for which the Canadian federal agencies sought modeller's advice was the optimal pathway to reopening after the lockdown, while avoiding a rebound in cases. The initial consensus results presented by modellers for the required stringency of measures was rejected, however, as being infeasible. It was therefore concluded that a second wave was inevitable. His research group subsequently approached the question as a stochastic optimization problem, with the objective to maximize population activity, subject to the constraint that ICU capacity would not be exceeded. In order to do this, the group's model was expanded to account for contact mixing. The impact of the emergence of new viral variants, and of vaccination, was incorporated next. Current ongoing work involves the addition of human behavior factors to the model, using deep learning techniques.

The final part of the session was a round-table discussion involving all the session speakers. The first topic of conversation was strengths, weaknesses and possible future directions for ensemble-based forecast modelling, a theme that had repeatedly come up both in this and earlier sessions. As a promising area for future development, Rebecca Borchering suggested making the weighting of ensemble models more ‘situational’ by quantifying which models perform better not just over some past time frame, but in specific phases (e.g., growth, decay) of a pandemic or epidemic. She also pointed to being able to report results of many models with a single voice as an important strength of the ensemble approach. Marc Baguelin cautioned that when many models in an ensemble are very similar and produce similar results, consideration needs to be given to how results can be weighted in a way that preserves diversity. Conversely, when models produce diverging results, he stated that it is important to understand what is driving the differences. Simon Cauchemez said that from the perspective of policymakers, ensembles are the best approach because they capture the diversity of opinion among modellers. At the same time, an ensemble made entirely of poorly-performing models will likely perform poorly itself, thus quantity of models alone is not sufficient. He also referred that countries that have a lot of modellers have an advantage in creating ensembles, whereas countries with fewer modellers would struggle. Finally, having one's model be just one of a larger group may be detrimental to the careers of trainees (graduate students, postdocs) because it takes away from the novelty of their work. Thorsten Lehr agreed that naively combining models ‘democratically’, with equal weights, is not desirable. On the topic of lower modelling capacity in some countries, he suggested that many models created in one country can readily be applied in others, but the bottleneck is lacking availability and standardization of data. Finally, Jianhong Wu mentioned the Ontario Modelling Consensus Table as a form of ensemble, and echoed Rebecca's point that decision-makers prefer to hear a single voice from modellers.

The next topic was how to best communicate results to decision-makers. For Marc Baguelin, in the case of the UK, the pre-existence of an avenue of communication between modellers and decision-makes, in the form of SPI-M-O, was an important advantage; this was then greatly expanded early in the pandemic. Nevertheless, the situation was far from perfect, UK decision makers became less responsive to advice from modellers over the summer of 2020, and a worst-case scenario produced by SPI-M-O, intended to be confidential, was leaked to the public. Rebecca Borchering stated that in the US, the Modelling Hub was helpful both in disseminating policy questions to modellers, and then in aggregating and communicating the results back to them. Jianhong Wu reported that in addition to the national modelling expert panel, the Public Health Agency of Canada also has its own in-house group of modellers; during the acute part of the Covid-19 pandemic, both groups would jointly make a single weekly report to the cabinet. Speaking to the situation in France, Simon Cauchemez said that along the path from modellers to policy-makers, detail would sometimes be lost, but subsequent efforts to address the problem by making communication more bi-directional and less hierarchical were overall successful. In Germany, Thorsten Lehr said, communication between modellers and the government underwent some changes with the transition to the new government, with some adjustments required. In his experience, politicians would sometimes selectively use only parts of modellers' advice to justify policy. At the same time, the voice of modellers in media became stronger over time. When making dashboards available to the general public, Thorsten Lehr emphasized that appropriately simplifying the interface is very important, as is communicating uncertainty in forecasts. And finally, he mentioned that it is key for modellers to understand policy-makers' available strategy options and constraints in order to be able to better address their needs.

## 7 Scenario planning and analysis

The session on scenario planning included six presentations illustrating not only the use of modelling for predicting future trends in disease evolution but also for assessing the impact of interventions aimed at mitigating disease progression.

### 7.1 Forecasting SARS-CoV-2 hospitalizations using EHR: application to Bordeaux hospital

Rodolphe Thiébaut shared his team's experience in leveraging hospital data warehouse to enhance Covid-19 forecasting in Bordeaux, France to inform the healthcare practitioners and the public on expected disease progression and, by doing so, supporting efficient decision making at a local level. This work highlighted the value of a robust hospital data warehouse to improve forecasting compared with forecasts based only on disease surveillance data (Ferté et al., 2022).

### 7.2 Hospital resources in time of epidemic crisis

In the second presentation, Pascal Crépey focused on ICU and bed capacity indicators. As one of the main issues during the Covid-19 pandemic was the ability of the health system to deal with the health needs generated by the disease, these indicators were used as a trigger for the implementation of more stringent social distancing measures. However, experience indicated that the health system was able to adapt to increase notably ICU capacity or to organize transfer of patients between hospitals which made hospital capacity more relative than initially viewed (Berger et al., 2022; Lefrant et al., 2021; Weiner-Lastinger et al., 2022). This questions the validity of such indicators as notably the extension of capacity might also involve a degradation of the medical care provided to patients. Other indicators such as gradient of increased fatality rates might more able to capture not only the quantity but also the quality of hospital capacity.

### 7.3 Scenario planning in the context of AIOLOS

Nicolas Lambert reported on the efforts done in the context of the AIOLOS project to develop scenario planning tools. The Covid-19 experience was used in the development of this tool to assess their capacity to anticipate the need for and expected impact of measures such as social distancing or vaccination in the medium term (6 months). This work highlights that questions for scenario planning are time-dependent and answers need to be informed by the change in disease environment (e.g., emergence of variants) that requires regular adaption to the tool used. He also indicated that in future developments, agent-based modelling as well as additional data sources will be explored to increase model flexibility and robustness.

### 7.4 Case introductions and their mitigation

Julien Arino focused his presentation on spatial dynamics and the effectiveness of travel restrictions measures during the Covid-19 pandemic. His analysis suggests a very limited value of such measures, except for remote areas not already affected by the disease (Hurford et al., 2023), as once the disease is introduced in a given location, the main driver of disease transmission is no longer imported cases but rather local transmission (Arino et al., 2021). In practice, travel restrictions are often implemented at a too late stage and without enough efficacy and more motivated by political considerations rather than expected public health benefits.

### 7.5 Decoding global spread dynamics modelling and scenario analysis

On a similar topic, Francesco Parino, provided comparable but more nuanced conclusions. He focused on metapopulation models incorporating global human mobility and recreating complex spreading patterns. Specifically, many of the results presented emanated from using the Global Epidemic and Mobility (GLEaM) model (Balcan et al., 2010). These models have played a key role in conducting scenario analyses, revealing that the main impact of travel restrictions is to delay the arrival of diseases, showcasing their restricted effectiveness in preventing the spread of epidemics. Simulations for influenza A/H1N1 and the 2014 Ebola outbreak showed a few weeks delay in case importations (Bajardi et al., 2011; Poletto, Gomes, et al., 2014). For Covid-19, initial travel restrictions reduced exportations from China but failed to prevent international seeding, and models showed the importance of the subsequent importations originating from regions different from the source of the epidemic (Pinotti et al., 2020). Conversely, synergistic effects arising from the coupling of travel restrictions with reductions in transmissibility have been elucidated (Chinazzi et al., 2020), with geographical nuances at the regional scale (Parino et al., 2021). He also underscored the significance of assessing delays resulting from travel restrictions in the context of response preparedness. Additionally, he acknowledged characteristics such as asymptomatic infections and the timing of symptom onset as crucial factors affecting the effectiveness of travel restrictions. In addition, to inform intervention policies, global metapopulation models have been utilized to create risk maps estimating importation probabilities for various outbreaks, including SARS-CoV-1 (Colizza et al., 2007), influenza A/H1N1 pandemic (Balcan et al., 2009), Middle East respiratory syndrome coronavirus (MERS-CoV) (Poletto, Pelat, et al., 2014), Poletto, Pelat, et al., 2014nd Ebola (Poletto, Gomes, et al., 2014). Assessments for Covid-19 calculated importation risks in Europe and correlated the risk with health-related preparedness in Africa (Gilbert et al., 2020; Pullano et al., 2020). Finally, he proposed coupling modelling with phylogeographic diffusion, enabling metapopulation model parameterization for seasonal influenza.

### 7.6 Agent-based modelling for short-term forecasting during emerging infectious diseases

Finally, Seyed Moghadas shared his experience in forecasting using agent-based modelling in Canada and the US and notably on methods for model calibration in real-time. He discussed the advantages and drawbacks of various methods: Ad-hoc calibration, which can be fast but often relies on a limited number of parameters and therefore not allowing the exploration of the entire parameter space; optimization techniques based on loss of functions, which allows a better exploration of the parameter space but do not necessarily inform on parameter uncertainty and can converge to a local rather than a global minimum; and Bayesian technique that is the most powerful but also the more demanding in terms of computer power which is challenging for real-time forecasting.

This session provided several illustrations on the value of scenario planning for not only inform on disease progression and helping decision making but also to assess the effectiveness of various interventions such as travel restrictions and vaccination, or to develop indicators such as importation risk which insights can be extrapolated to other diseases or situations. It also highlighted the difficulties of the exercise that not only requires accessing sufficient data both in terms of quantity and quality and this with limited delays between the observation and its use in modelling analyses. The time and effort for analyzing data and calibrating models should also not be overlooked to avoid unsatisfactory trade-off between the quality of the method used and the time pressure for generating results. Finally, scenario planning rests on disease dynamics but also on human behavior which remains hard to predict but is an important aspect to factor in scenario planning exercises.

## 8 Integrating insights across sessions

The combination of early warning systems and scenario planning exemplifies the necessity for adaptable, multi-source data integration strategies. Lessons from early warning systems underscore the potential for more dynamic and responsive scenario planning exercises, emphasizing the critical role of timely and diverse data sources in predicting and managing disease outbreaks.

Innovations in data integration and modelling, such as those demonstrated through wastewater epidemiology, offer profound insights for pandemic preparedness. These advances not only facilitate early detection and monitoring but also contribute valuable knowledge for public health policy and scenario planning. The ability to adapt solutions identified in modelling SARS-CoV-2 to other pathogens with pandemic potential highlights the universality and flexibility of these modelling challenges and solutions across different infectious diseases.

Interdisciplinary approaches are needed for advancing infectious disease modelling. Combining expertise from epidemiology, data science, behavioral science, and public health policy enriches our understanding and management of diseases. The different sessions underscored the importance of transparency, public engagement, and ethical considerations in data collection and model development. These principles are vital cross-cutting themes that ensure the reliability and acceptability of modelling efforts.

## 9 Conclusion

A central theme throughout the workshop was the critical role of multisource data utilization. While recognizing the continued significance of traditional surveillance data collection, the participants acknowledged the substantial benefits of integrating non-traditional approaches (mobility, pharmacy consumption, wastewater monitoring, social media). A holistic data approach is crucial in understanding and predicting disease spread, especially in the face of swift changes in behavior, interventions, the emergence of new variants, and evolving severity (Perez-Guzman et al., 2023). Participants emphasized the importance of incorporating human behavioral data into Covid-19 modelling to improve the accuracy of transmission pattern predictions. However, challenges arise due to the inherent difficulty in tracking real-time human behavior. The expansion of new approaches for social listening and infodemic management was highlighted. Such strategies are essential for designing public health measures and social interventions during future pandemics, taking community knowledge, attitudes, and practices into account.

It was also underlined the various challenges faced by Covid-19 modelling efforts, ranging from poor data quality to the unpredictability of policy changes and human behavior. These issues underscore the need for adaptable and robust modelling frameworks. A promising avenue discussed was the application of tools and models developed during the pandemic for other respiratory viruses with pandemic potential, suggesting potential wider applications of these innovations.

A strong response to pandemics built upon robust seasonal influenza systems was also emphasized, coupled with the need to maintain capabilities during non-pandemic periods to be able to quickly react in case of new threats.

The importance of open modelling collaborations was stressed, especially those coordinated between multiple teams, public health agencies and also public-private partnerships. These collaborations are instrumental in creating effective systems, encouraging model development and comparison, and fostering a community committed to open science. The development of modelling hubs in the US and Europe, which collate projections from various models, was noted as particularly important during crises. These hubs facilitate coordinated responses in times of high uncertainty and have significantly impacted public health decision-making (Borchering et al., 2023). The continuation and support of these hubs during peacetime was emphasized, ensuring readiness and commitment from participating teams.

A notable gap identified was the lack of a common international framework for epidemiological data sharing. Establishing such a framework, along with international surveillance systems, is vital. Investment in infrastructure for data sharing was urged as a priority for governments, extending beyond the pandemic.

Finally, the workshop highlighted the responsibility of modellers in ensuring their work is accurately interpreted. During the Covid-19 pandemic, there were times when modelling results were sensationalized or politicized, leading to misunderstandings and potentially serious consequences. Modelling research should be communicated to the public by expert translators who can contextualize and clarify the data appropriately. Therefore, modellers must be proactive in facilitating the appropriate interpretation of their findings, considering the uncertainty and fear that often accompany public health crises.

Table 2 provides an overview of future directions and unanswered questions in infectious disease modelling, highlighting the path forward and the most promising areas for impactful research.

**Table 2:** Future directions and questions to be answered.

Path forward:
Infectious disease modelling can support infectious disease control owing to predicting the magnitude of disease epidemics, elucidating transmission dynamics, assessing the effectiveness of interventions or policies, and warning or forecasting prior to outbreaks. The integration of artificial intelligence, machine learning, and big data analytics is pivotal in advancing real-time surveillance, early detection, and predictive modelling.
The evolving role of digital epidemiology, through the use of social media and mobility data, has significantly transformed the landscape of infectious disease monitoring and prediction. This transformation is part of a broader shift towards leveraging digital tools and data sources to augment traditional epidemiological methods, offering new avenues for real-time surveillance, early warning systems, and public health interventions. The integration of these data sources presents a promising future, enabling more dynamic and responsive public health surveillance and intervention strategies. However, it also raises significant ethical, privacy, and methodological challenges that must be addressed. Ensuring data accuracy, representativeness, and privacy requires careful consideration and innovative solutions.
Unanswered questions:
Data quality and accessibility: How can the global health community address the challenges related to the quality, standardization, and sharing of health data? What frameworks can be developed to ensure data are not only accessible but also actionable for public health decisions?
Models transparency and interpretability: With the increasing use of complex models, what strategies can ensure these models remain transparent and interpretable to policymakers, stakeholders, and the general public?
Behavioral dynamics: How can models better incorporate human behavior and its impact on disease transmission dynamics, especially in light of changing societal norms and misinformation?
Global collaboration: How can the global health community foster stronger collaborations and knowledge exchange to prepare for and respond to future pandemics? What roles do public-private partnerships play in this?

## Disclaimers

Any use of trade, firm, or product names is for descriptive purposes only and does not imply endorsement by the US Government. The findings and conclusions in this report are those of the authors and do not necessarily represent the views of the Centers for Disease Control and Prevention or the National Institutes of Health.

## CRediT authorship contribution statement

**Marta C. Nunes:** Writing – original draft, Conceptualization. **Edward Thommes:** Writing – original draft, Conceptualization. **Holger Fröhlich:** Writing – review & editing, Validation, Conceptualization. **Antoine Flahault:** Writing – review & editing, Visualization, Conceptualization. **Julien Arino:** Writing – review & editing, Visualization. **Marc Baguelin:** Writing – review & editing, Validation. **Matthew Biggerstaff:** Writing – review & editing, Visualization. **Gaston Bizel-Bizellot:** Writing – review & editing, Visualization. **Rebecca Borchering:** Writing – review & editing, Visualization. **Giacomo Cacciapaglia:** Writing – review & editing, Visualization. **Simon Cauchemez:** Writing – review & editing, Visualization. **Alex Barbier--Chebbah:** Writing – review & editing, Visualization. **Carsten Claussen:** Writing – review & editing, Visualization. **Christine Choirat:** Writing – review & editing, Visualization. **Monica Cojocaru:** Writing – review & editing, Visualization. **Catherine Commaille-Chapus:** Writing – original draft. **Chitin Hon:** Writing – review & editing, Visualization. **Jude Kong:** Writing – review & editing, Visualization. **Nicolas Lambert:** Writing – review & editing, Visualization. **Katharina B. Lauer:** Writing – review & editing, Visualization. **Thorsten Lehr:** Writing – review & editing, Visualization. **Cédric Mahe:** Writing – review & editing, Visualization, Conceptualization. **Vincent Marechal:** Writing – review & editing, Visualization. **Adel Mebarki:** Writing – review & editing, Visualization. **Seyed Moghadas:** Writing – review & editing, Visualization. **Rene Niehus:** Writing – review & editing, Visualization. **Lulla Opatowski:** Writing – review & editing, Visualization. **Francesco Parino:** Writing – review & editing, Visualization. **Gery Pruvost:** Writing – review & editing, Visualization. **Andreas Schuppert:** Writing – review & editing, Visualization. **Rodolphe Thiébaut:** Writing – review & editing, Visualization. **Andrea Thomas-Bachli:** Writing – review & editing, Visualization. **Cecile Viboud:** Writing – review & editing, Visualization. **Jianhong Wu:** Writing – review & editing, Visualization, Conceptualization. **Pascal Crépey:** Writing – review & editing, Visualization, Conceptualization. **Laurent Coudeville:** Writing – original draft, Conceptualization.

## Declaration of competing interest

MCN reports grants from the Bill & Melinda Gates Foundation, European & Developing Countries Clinical Trials Partnership, Pfizer, AstraZeneca, and Sanofi; and consultation fees outside the work reported here from Sanofi. TL received funding from the Government of the Saarland for the maintenance and development of the COVID Simulator. SMM reports advisory roles for Janssen Canada and Sanofi for cost-effectiveness of their vaccine products, and received consultation fees outside the work reported here. JW acknowledges support from NSERC-Sanofi Industrial Research Chair program and the NSERC Alliance program. PC reports consulting fees from Sanofi, Pfizer, and Seqirus. Fraunhofer-Institute declares various national and international public and private grants which are in line with its status as a non-for-profit research organization.
